# Intermittent Fasting Aggravates Lupus Nephritis through Increasing Survival and Autophagy of Antibody Secreting Cells in MRL/lpr Mice

**DOI:** 10.3390/ijms21228477

**Published:** 2020-11-11

**Authors:** Seung-Min Hong, Jaeseon Lee, Se Gwang Jang, Youngseok Song, Minjun Kim, Jennifer Lee, Mi-La Cho, Seung-Ki Kwok, Sung-Hwan Park

**Affiliations:** 1 Rheumatism Research Center, Catholic Research Institute of Medical Science, College of Medicine, The Catholic University of Korea, Seoul 06591, Korea; dkask0809@catholic.ac.kr (S.-M.H.); phlox@catholic.ac.kr (J.L.); elite@catholic.ac.kr (S.G.J.); suky0215@catholic.ac.kr (Y.S.); rlaalswns102@naver.com (M.K.); poohish@catholic.ac.kr (J.L.); iammila@catholic.ac.kr (M.-L.C.); seungki73@catholic.ac.kr (S.-K.K.); 2 Division of Rheumatology, Department of Internal Medicine, Seoul St. Mary’s Hospital, College of Medicine, The Catholic University of Korea, Seoul 06591, Korea

**Keywords:** systemic lupus erythematosus, MRL/lpr, fasting, autophagy, plasma cells

## Abstract

Systemic lupus erythematosus (SLE) is an autoimmune disease in which the main contributors to organ damage are antibodies against autoantigens, such as double-stranded DNA (dsDNA). Calorie restriction and intermittent fasting (IF) have been shown to improve autoimmune disease symptoms in patients and animal models. Here, we tested the hypothesis that IF might improve symptoms in MRL/lpr mice, which spontaneously develop an SLE-like disease. Groups of mice were fed every other day (IF) or provided food ad libitum (controls), and various lupus-associated clinicopathological parameters were analyzed for up to 28 weeks. Contrary to expectations, anti-dsDNA antibody levels, immune complex deposition in the kidney, and glomerular injury were higher in the IF group than the control group, although there were no differences in spleen and lymph node weights between groups. Proteinuria was also worsened in the IF group. IF also increased the abundance of B cells, plasmablasts, and plasma cells and elevated autophagy in plasma cells in the spleen and lymph nodes. Secretion of anti-dsDNA antibody by splenocytes in vitro was reduced by chloroquine-induced inhibition of autophagy. These results suggest that IF exacerbates lupus nephritis in MRL/lpr mice by increasing autoantibody immune complex formation.

## 1. Introduction

Systemic lupus erythematosus (SLE) is an autoimmune disease that can affect many organs, resulting in splenomegaly, lymphadenopathy, nephritis, and/or skin damage [1]. Immune complexes formed by binding of autoantibodies to antigen are thought to be a major mediator of SLE [1]. In particular, anti-double-stranded DNA (anti-dsDNA) antibodies are found in the sera of up to 70% of SLE patients, and their presence is 95% specific for SLE, making them an important disease hallmark [1]. Correlations between anti-dsDNA antibodies titers and SLE disease activity further suggest that these autoantibodies play a pathogenic role in SLE [2]. Although current treatments for SLE have dramatically improved survival, a cure, or even long-term relief of symptoms, remain elusive for most patients [3].

B cells play a central role in the adaptive immune response. Engagement of the B cell receptor for antigen induces B cell activation and differentiation into germinal center B cells, plasma cells, and memory B cells. In SLE patients, B cells are considered to be overactive [4,5,6,7], and defective regulation of B cell activation has been demonstrated in several animal models of SLE [7,8,9,10]. Lupus nephritis, which correlates with anti-dsDNA titers, is one of the most common symptoms of SLE [2]. Notably, treatment of SLE patients with drugs targeting B cells has achieved some success [11].

Diet control through calorie restriction (CR) or intermittent fasting (IF) has been shown to improve symptoms and pathological features of some autoimmune diseases [12,13,14]. However, chronic CR may have harmful health effects, such as excessive weight loss and increased susceptibility to pathogen infection [15,16]. IF is a favorable alternative method to CR of reducing dietary intake because it is less restrictive, which leads to better compliance [17].

IF is involved in immune regulation in several ways. For example, IF increases regulatory T cells in WT mice or (NZB × NZW) F1 lupus-prone mice [18]. In the ischemic stroke, IF downregulates inflammasome activity by regulating NF-κB and MAPK which are transcription factors involved in the production of inflammatory cytokines [19]. In the multiple sclerosis animal model, IF decreases IFN-γ, TNF-α, and T cell proliferation [20]. However, poly(I:C)-treated mice shows increasing of circulating levels of cytokines (TNF-α, MCP-1, IL-6, IL-10, IFN-α, and IFN-γ) in IF group [21]. In lupus, several cytokines are increased (IL-6, IL-17, IFN-γ, etc.) or decreased (IL-2) [22]. Whether IF regulates these cytokines in lupus is not yet known.

In MRL/lpr mice, CR reduces serum anti-dsDNA antibody concentration, splenomegaly, and lymph node (LN) size [13]. Thus, we hypothesized that IF may also alleviate the phenotypes of spontaneous SLE in these mice. We compared various clinical and cellular parameters associated with SLE development in groups of mice allowed access to food every other day or ad libitum. Surprisingly, we found that B cell activation, autoantibody production, and lupus nephritis severity were increased in the IF group compared with the control group. Our results therefore suggest that, despite having beneficial effects in other models of autoimmune disease, IF may be detrimental for patients with SLE.

## 2. Results

### 2.1. IF Increases Serum Anti-dsDNA Antibody Concentration and Aggravates Lupus Nephritis in MRL/lpr Mice

**Figure 1:**
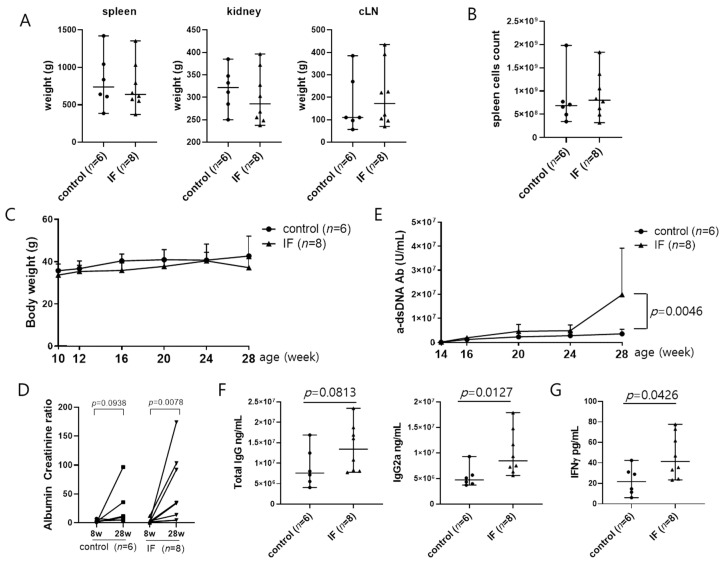
Intermittent fasting elevates autoantibody secretion in MRL/lpr mice. (**A**) Spleen, cervical lymph node (cLN), and kidney weights in mouse groups at 28 weeks of age. (**B**) Total cell counts in mouse spleens collected at 28 weeks. (**C**) Body weights between 10 and 28 weeks. (**D**) Albumin to creatinine ratio assessed in urine samples collected at 8 and 28 weeks. (**E**) Serum anti-dsDNA IgG levels measured between 14 and 28 weeks. (**F**,**G**) Serum total IgG, IgG2a, and IFN-γ levels measured at 28 weeks. Data are presented as the median with range or mean ± SD and are representative of three independent experiments. Data were analyzed using Mann–Whitney U test (**A**,**B**,**F**,**G**), Wilcoxon test (**D**), or two-way ANOVA (**C**,**E**).

**Figure 2:**
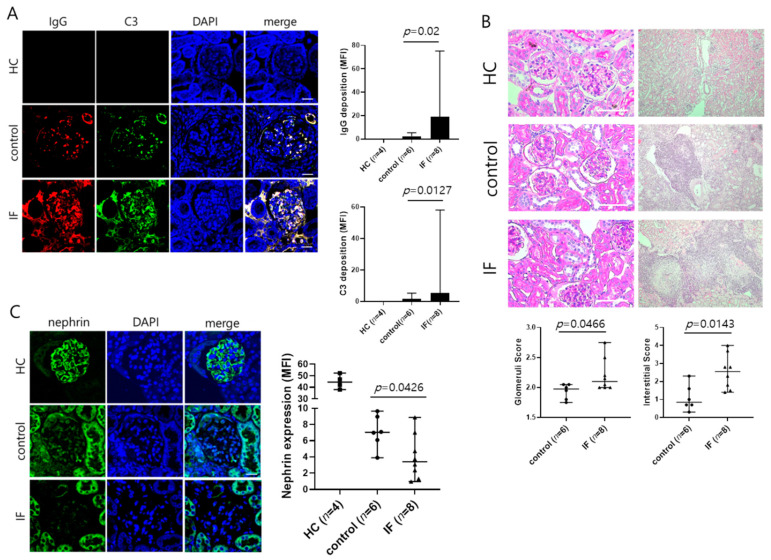
Intermittent fasting increases immune complex deposition and aggravates glomerular injury. (**A**) Left: Representative immunofluorescence micrographs of kidney sections stained for IgG and C3. Right: Mean fluorescence intensity (MFI) of IgG and C3 staining. Scale bar: 20 μm. HC = healthy control. (**B**) Upper: Representative photomicrographs of PAS-stained or H&E stained sections of kidney. Lower: Histopathologic scores of glomerular injury (PAS staining) and interstitial immune cell infiltration (H&E staining). Scale bar: 100 μm. (**C**) Left: Representative immunofluorescence micrographs of kidney sections stained for nephrin. Right: MFI of nephrin staining. Scale bar: 20 μm. Data are presented as the median with range and are representative of three independent experiments. Data were analyzed using Mann–Whitney U test.

Two groups of female MRL/lpr mice aged 10 weeks were subjected to IF (*n* = 8) or fed ad libitum (control, *n* = 6) and monitored until they were 28 weeks of age. No differences between the IF and control groups were observed in spleen, cLN, kidney, or body weights or in the total number of splenocytes (Figure 1A–C). However, proteinuria was worsened, and anti-dsDNA antibody and IgG2a concentration of serum were significantly increased in the IF group (Figure 1D–F). IF also increased production of IFN-γ in serum in the IF group (Figure 1G). Immunostaining of kidney sections revealed higher IgG and C3 deposition in the glomeruli of the IF group compared with the control group (Figure 2A) that was accompanied by more extensive glomerular injury and interstitial inflammation (Figure 2B). However, expression of nephrin, a podocyte marker, was decreased in the glomerulus of the IF group compared with the control group (Figure 2C). These results suggest that IF increased autoantibody immune complex deposition in the glomeruli, thereby exacerbating lupus nephritis.

### 2.2. IF Increases the Abundance of Spleen Plasmablasts and Plasma Cells

**Figure 3:**
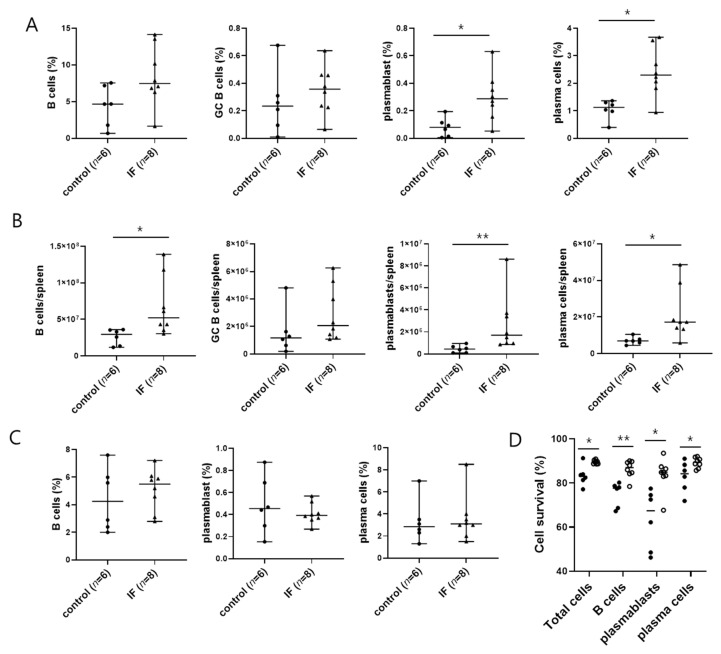
Intermittent fasting increases the proportion and number of plasmablasts and plasma cells in spleen from MRL/lpr mice. (**A**) Percentages of spleen B cells (FVD− CD19+ cells), germinal center B cells (FVD− CD19+ GL7+ cells), plasmablasts (FVD− CD90.2− CD19+ CD138+ cells), and plasma cells (FVD− CD90.2− CD19− CD138+ cells). (**B**) Total numbers of spleen B cells, plasmablasts, and plasma cells. (**C**) Percentages of bone marrow B cells (FVD− CD19+ cells), plasmablasts (FVD− CD19+ CD138+ cells), and plasma cells (FVD− CD19− CD138+ cells). (**D**) Percentage of FVD− cells among total spleen cells, B cells, plasmablasts, and plasma cells in the spleens of control mice (closed circles; *n* = 6) and IF mice (open circles; *n* = 8). Data are presented as the median or median with range and are representative of three independent experiments. Data were analyzed using Mann–Whitney U test. * *p* < 0.05; ** *p* < 0.01.

To determine the mechanism of elevated anti-dsDNA antibody production in MRL/lpr mice subjected to IF, we isolated spleen and bone marrow cells and analyzed the subpopulations by flow cytometry. The proportion and number of plasmablasts and plasma cells (Figure 3A,B) as well as the number of B cells (Figure 3B) were increased in the spleens of IF mice compared with control mice; however, there were no differences in the number or proportion of germinal center B cells (Figure 3A,B). In contrast to the spleen, IF had no effect on B cell subpopulations in the bone marrow (Figure 3C). Furthermore, IF had no effect on either the absolute number or ratio of follicular helper T cells (TFH) and follicular regulatory T cells, which regulate B cell differentiation (data not shown). We also investigated the difference in cell viability between spleen cells in the control and IF group. Surprisingly, the percentages of viable total spleen cells, B cells, plasmablasts, and plasma cells were higher in the IF group than the control group (Figure 3D). These results suggest that the observed increase in serum anti-dsDNA antibody levels in IF mice was caused by increases in the survival rates and numbers of B cells, plasmablasts, and plasma cells.

### 2.3. IF Elevates Autophagy in Plasma Cells

**Figure 4:**
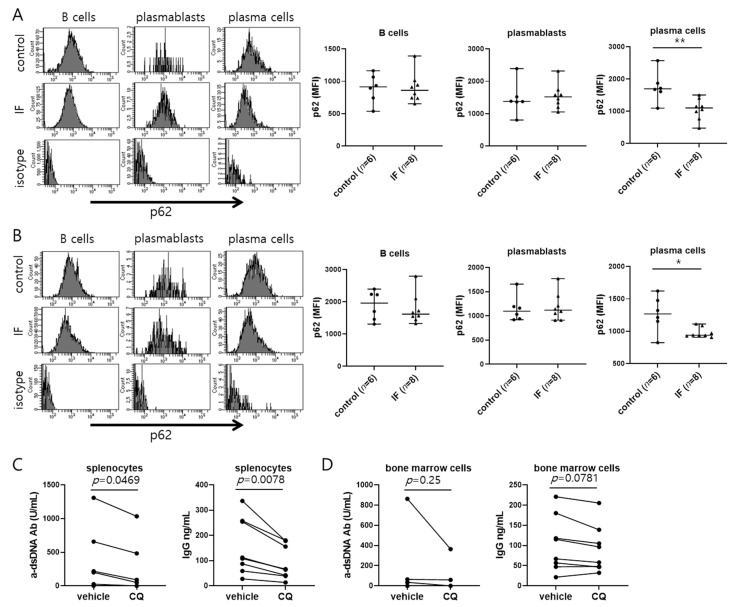
Intermittent fasting increases autophagy in the plasma cells of MRL/lpr mice and autophagy inhibitor downregulates the antibodies secretion. (**A**,**B**) Left panel, representative histograms comparing p62 expression gated for B cells, plasmablasts, and plasma cells in spleen cells (**A**) and bone marrow cells (**B**). Right panel, cumulative data of flow cytometry for p62 expression with mean fluorescence intensity (MFI). Gating strategies for spleen cells were B cells (FVD− CD19+ cells), plasmablasts (FVD− CD90.2− CD19+ CD138+ cells), and plasma cells (FVD− CD90.2− CD19− CD138+ cells). Gating strategies for bone marrow cells were B cells (FVD− CD19+ cells), plasmablasts (FVD− CD19+ CD138+ cells), and plasma cells (FVD− CD19− CD138+ cells). (**C**,**D**) Anti-dsDNA and total IgG concentrations in supernatants of spleen cells (**C**) and bone marrow cells (**D**) (*n* = 8) incubated for 24 h with 50 μM chloroquine (CQ). Data are presented as the median with range and are representative of three independent experiments. Data were analyzed using Mann–Whitney U test (**A**,**B**) or Wilcoxon test (**C**,**D**). * *p* < 0.05; ** *p* < 0.01.

IF is known to induce the cellular recycling pathway of autophagy [23], which plays a crucial role in cell survival [24] and is also required for B cell differentiation into plasma cells, maintenance of antibody secretion, and plasma cell survival [25,26]. We analyzed the effect of IF on autophagy in B cell subpopulations by flow cytometric staining of p62, which is degraded via autophagy and is therefore a measure of the autophagy rate [27]. We found that p62 levels were lower in plasma cells from the spleen and bone marrow of mice in the IF group compared with the control group (Figure 4A,B), indicative of an elevated rate of autophagy. To investigate whether increased autophagy may contribute to an increase in serum anti-dsDNA antibody levels in mice subjected to IF, we cultured splenocytes and bone marrow cells in vitro in the presence or absence of an autophagy inhibitor, chloroquine [28]. Indeed, treatment with chloroquine for 24 h reduced anti-dsDNA and IgG secretion by cells from IF group (Figure 4C,D). These results suggest that the higher serum autoantibody concentration in mice in the IF group was at least partly attributable to an increase in autophagy in plasma cells.

### 2.4. IF Increases the Expression of Antiapoptotic Factors in Spleen

**Figure 5:**
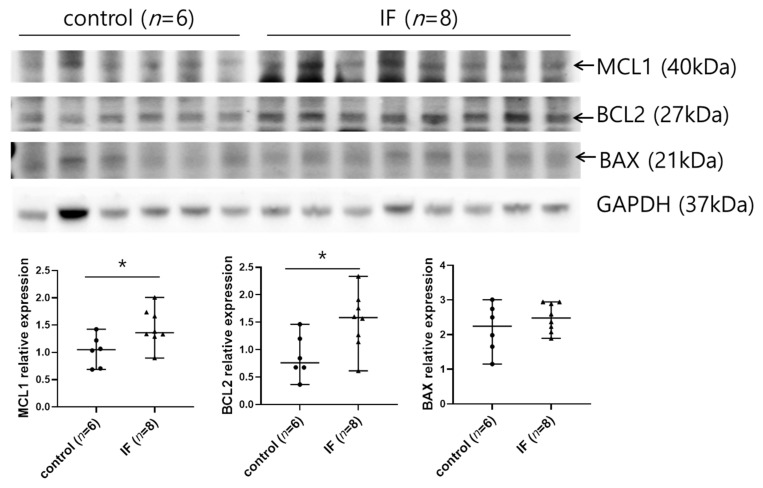
Analysis of apoptosis-related protein expression in spleen. Upper: Western blot analysis of MCL1, BCL2, and BAX in lysates of spleen tissues from mice in the control and IF groups. Lower: Quantification of MCL1, BCL2, and BAX levels relative to that of GAPDH in the control and IF groups. Data are presented as the median with range and are representative of three independent experiments. Data were analyzed using Mann–Whitney U test. * *p* < 0.05.

Because autophagy was only increased in plasma cells, but not B cells or plasmablasts, from mice subjected to IF (Figure 4A,B), we speculated that other pathways could contribute to the elevated survival of B cells and plasmablasts in these mice. We analyzed levels of the apoptosis-related BCL2 family of proteins in the spleens of IF or control mice by western blotting. Levels of the antiapoptotic proteins MCL1 and BCL2 were higher in spleen cells from the IF group than the control group, whereas IF did not affect expression of the proapoptotic protein BAX (Figure 5). Given that MCL1 and BCL2 regulate the survival and development of B cells, plasmablasts, and plasma cells [29], these results suggest that increased expression of MCL1 and BCL2 could promote B cell survival and formation of plasma cells, ultimately contributing to the increase in autoantibody secretion in mice subjected to IF.

## 3. Discussion

The results of the present study show that serum autoantibody levels were increased and glomerular injury and interstitial inflammation in the kidney were exacerbated in MRL/lpr mice subjected to IF compared with their fed counterparts. IF also increased the survival and abundance of B cells, plasmablasts, and plasma cells in the spleen, but did not affect germinal center B cells, follicular T helper cells, or follicular regulatory T cells. Autophagy was elevated in plasma cells, but not B cells or plasmablasts, from mice in the IF group, and expression of antiapoptotic factors was also upregulated in spleen from mice subjected to IF. Collectively, these results demonstrate that IF exacerbates disease in MRL/lpr mice by increasing the number and rate of autophagy in antibody-secreting plasma cells, resulting in elevated autoantibody secretion and immune complex deposition in the glomerulus.

Our results indicate that IF and CR do not have the same effect on disease activity in MRL/lpr mice [13]. CR has been shown to reduce spleen, LN, and body weights and to suppress anti-dsDNA antibody production in MRL/lpr mice [13], whereas we found that IF had no effect on body or organ weights and actually increased anti-dsDNA antibody levels. While the exact reason for this difference is unknown, one possible explanation is that CR, but not IF, has a striking effect on body weight in this mouse strain. Indeed, MRL/lpr mice subjected to CR for 2 months had about half the body weights of the control mice [13]. These differences are consistent with the known effect of nutrient intake and body weight loss on the immune response [30,31]. Both CR and IF have been shown to induce autophagy in cells from MRL/lpr mice [23], but CR alone induced a reduction in spleen and LN size that may have contributed to the decline in anti-dsDNA antibody production [13]. However, more research is needed to determine the exact reason for the different effects of CR and IF in MRL/lpr mice.

Autophagy is closely related to the immune response. Autophagy participates in antigen presentation of APC, digestion of pathogen, and T cells proliferation and survival [32]. Furthermore, autophagy has a well-established role in B cell differentiation, plasma cell survival, and antibody secretion [24,25]. Thus, it is reasonable to assume that IF-induced increases in autophagy might affect plasmablast and plasma cell numbers and survival. In the present study, however, autophagy was not increased in B cells or plasmablasts of mice subjected to IF, indicating that further work will be necessary to clarify the link between IF and autophagy in the B cell lineage in MRL/lpr mice. There are many reports suggesting that autophagy is involved in lupus pathogenesis. For instance, several SNPs in the Atg5 gene give susceptibility to lupus [33]. In activated lymphocyte-derived DNA-induced lupus animal model, when macrophages were removed and then macrophages were transplanted again, lupus did not develop in the group transplanted with macrophages transfected with shRNA against beclin1 [34]. In a Tlr7 transgenic mice model of lupus induced by overexpression of Toll-like receptor 7, secretion of autoantibodies is reduced when autophagy is suppressed through knockout of the autophagy protein ATG5 in B cells [35]. In our study, we detected an increase in autophagy in plasma cells from mice in the IF group, and inhibition of autophagy in vitro inhibited autoantibody secretion by spleen cells from IF group, which suggests that IF-induced increases in autophagy can contribute to elevated autoantibody secretion.

IF affects production of cytokines in several disease models [19,20,21]. In our experimental results, only IFN-γ was increased in the sera of the IF group and there was no difference in the expression of other cytokines (IL-2, IL-4, IL-6, IL-10, IL-17, TNF-α, BAFF; data not shown). IFN-γ plays an important role in the pathogenesis in MRL/lpr mice [36]. Therefore, increase of IFN-γ in the IF group may have an effect on worsening lupus symptoms. Furthermore, because IFN-γ enhances secretion of IgG2a, it may have an effect on the increase of IgG2a in the IF group [37,38]. IFN-γ also affects the viability and differentiation of B cells. IFN-γ slightly increases the survival of B cells [39]; however, when stimulated with IL-4, it decreases survival [40]. In addition, IFN-γ is known to increase germinal center B cells in lupus animal model [41], but there was no difference in our study. Further research is needed to clarify whether the increased IFN-γ regulated the viability and differentiation of B cells in the IF group.

IF is known to induce apoptosis or antiapoptosis [42,43,44,45]. In the present study, we detected increased expression of the antiapoptotic proteins MCL1 and BCL2 in the spleens of mice subjected to IF. These proteins regulate the survival of B cells and formation of plasma cells [29], raising the possibility that IF-induced inhibition of apoptosis is responsible for the increased survival rates of B cells, plasmablasts, plasma cells, and total spleen cells. Further experiments are needed to understand the molecular mechanisms by which IF increases MCL1 and BCL2 in MRL/lpr mice.

In conclusion, we found that IF exacerbated SLE-like disease activity in MRL/lpr mice. IF resulted in elevated survival and autophagy rates of plasma cells in vivo, and autophagy was shown to contribute to autoantibody production in vitro. IF also increased autoantibody immune complex deposition in the glomerulus of MRL/lpr mice, thereby aggravating lupus nephritis. We conclude that IF may be detrimental for patients with SLE.

## 4. Materials and Methods

### 4.1. Animals

Female MRL/lpr mice were purchased from SLC Inc. (Hamamatsu, Shizuoka, Japan) and randomized into a control group (*n* = 6) and an intermittent fasting group (IF; *n* = 8). MRL/lpr mice were sacrificed at 28 weeks of age. Control mice had unrestricted access to food. Mice in the IF group were fed every other day (food pellets were provided or removed at 9 am) between 10 and 28 weeks of age. Male C57BL/6 mice were purchased from OrientBio (Sungnam, Korea). C57BL/6 mice were sacrificed at 12 weeks of age and used as healthy control (*n* = 4). All procedure of animal research were provided in accordance with the Laboratory Animals Welfare Act, the Guide for the Care and Use of Laboratory Animals and the Guidelines and Policies for Rodent experiment provided by the IACUC (Institutional Animal Care and Use Committee) in School of Medicine, The Catholic University of Korea (approval number: CUMS-2020–0071-03 and CUMS-2020–0031-02).

### 4.2. Measurement of Serum Antibodies and Cytokine

Anti-dsDNA antibody levels were measured in 1:5000 dilutions of sera by ELISA (Alpha Diagnostics, San Antonio, TX, USA) according to the manufacturer’s instructions. Total IgG and IgG2a levels were measured in 1:100,000 dilutions of sera by ELISA (Bethyl Laboratories, Montgomery, TX, USA) according to the manufacturer’s instructions. IFN-γ levels were measured in 1:10 dilutions of sera by ELISA (R&D Systems, Minneapolis, MN, USA) according to the manufacturer’s instructions.

### 4.3. Measurement of Urine Albumin to Creatinine Ratio

Spot urine samples were collected by urinary bladder massage of each mouse at 8 and 28 weeks of age. Urine albumin and creatinine concentrations were measured using a mouse albumin ELISA assay (Bethyl Laboratories, Montgomery, TX, USA) and a creatinine assay (R&D Systems, Minneapolis, MN, USA), respectively, according to the manufacturer’s directions. Urine albumin excretion was expressed as the ratio of urine albumin to creatinine.

### 4.4. Immunofluorescence Microscopy

Sections of kidney tissues were incubated at 4 °C overnight with anti-mouse IgG (BioLegend, San Diego, CA, USA), anti-mouse C3 (Abcam, Cambridge, UK), or anti-mouse nephrin (Abcam, Cambridge, UK), washed, and incubated with anti-rat, anti-rabbit, or anti-mouse secondary antibodies conjugated to Alexa Fluor 488 or Alexa Fluor 594 for 2 h. Nuclei were stained with 4′,6-diamidino-2-phenylindole (DAPI; Invitrogen/Thermo Fisher Scientific, San Diego, CA, USA). Control sections were treated in the same manner except that rat, rabbit, or mouse IgG were used in place of the primary antibodies. Confocal microscopy was performed and images were acquired using an LSM 800 confocal microscope (Zeiss, Oberkochen, Germany). Ten renal glomeruli areas were examined and averaged.

### 4.5. Histologic Assessment of Kidney

Sections of kidney tissues were stained with periodic acid–Schiff (PAS) reagent (Abcam, Cambridge, UK) or Hematoxylin and Eosin (H&E) and analyzed using a digital camera (MedICAM-k; COMART system, Seoul, Korea) attached to an Olympus BX41 microscope. Kidney pathology was evaluated using the standard lupus nephritis classification system [46].

### 4.6. Flow Cytometry

Spleens were minced in RPMI 1640 medium (Gibco, Carlsbad, CA, USA) and filtered through a 40-μm cell strainer to prepare single-cell suspensions. Tibias were dissected under sterile conditions to expose the bone marrow cavity, which was then rinsed with RPMI 1640 medium. Cells were surface-stained with eFluor780-fixable viability dye (FVD) (eBioscience, Carlsbad, CA, USA), Pacific Blue-conjugated anti-CD90.2 (BioLegend, San Diego, CA, USA), allophycocyanin (APC)-conjugated anti-CD19 (BioLegend, San Diego, CA, USA), PerCP-Cy5.5-conjugated GL7 (BioLegend, San Diego, CA, USA), and/or PE-conjugated anti-CD138 (BD Biosciences, San Jose, CA, USA) antibodies. The cells were then fixed, permeabilized, and incubated with anti-p62 antibody (Abcam, Cambridge, UK) or isotype antibody (Thermo Fisher Scientific, San Diego, CA, USA) followed by an Alexa Fluor 488-conjugated secondary antibody (Invitrogen/Thermo Fisher Scientific, San Diego, CA, USA). Cells were analyzed using a BD LSRII Fortessa flow cytometer (BD Biosciences, San Jose, CA, USA).

### 4.7. Chloroquine Treatment of Cultured Cells

Splenocytes or bone marrow cells from the IF group were resuspended in RPMI 1640 medium (Gibco, Carlsbad, CA, USA) supplemented with 10% fetal bovine serum and 100 U/mL penicillin/streptomycin. The cells were seeded in 24-well plates at 1 × 10^6^ cells/well and incubated for 24 h with or without chloroquine 50 μM (Sigma-Aldrich, St Louis, MO, USA). The culture supernatants were then collected for measurement of anti-dsDNA antibody and total IgG concentration as described above.

### 4.8. Western Blot Analysis

Spleen samples were lysed in RIPA buffer containing Halt protease/phosphatase inhibitor cocktail (Thermo Fisher Scientific, San Diego, CA, USA) and incubated for 45 min at 4 °C. Samples of lysate equivalent to 20 μg of protein were separated by 10% or 12% sodium dodecyl sulfate–polyacrylamide gel electrophoresis, and proteins were transferred to PVDF membranes (Bio-Rad, Hercules, CA, USA). The membranes were blocked with Bovine Serum Albumin (Gibco, Carlsbad, CA, USA) and incubated with the following primary antibodies; anti-MCL1 (Proteintech, Chicago, IL, USA), anti-BCL2 (Bioss Biotechnology, Beijing, China), anti-BAX (Bioss Biotechnology), or anti-glyceraldehyde 3-phosphate dehydrogenase (GAPDH; Abcam, Cambridge, UK) for 15 h at 4 °C. The membranes were washed again and incubated with horseradish peroxidase-conjugated goat anti-rabbit IgG, goat anti-mouse IgG (both Thermo Fisher Scientific, San Diego, CA, USA), or mouse anti-goat IgG (Santa Cruz Biotechnology, Dallas, TX, USA). Bound proteins were visualized using SuperSignal West Pico Chemiluminescent substrate (Thermo Fisher Scientific, San Diego, CA, USA), and the membranes were analyzed using an Amersham Imager 600 (GE Healthcare, Chicago, IL, USA).

### 4.9. Statistical Analysis

Statistical analyses were performed using GraphPad Prism v.8.0 software (GraphPad, San Diego, CA, USA). Statistical significance was determined by the Mann–Whitney U test, by Wilcoxon test, or by two-way ANOVA with Bonferroni post hoc test. *p* < 0.05 was considered statistically significant.
